# Comparative Analysis of the Chalcone-Flavanone Isomerase Genes in Six *Citrus* Species and Their Expression Analysis in Sweet Orange (*Citrus sinensis*)

**DOI:** 10.3389/fgene.2022.848141

**Published:** 2022-04-12

**Authors:** Quan Wan, Tingting Bai, Minmin Liu, Ying Liu, Yating Xie, Tao Zhang, Min Huang, Jinlian Zhang

**Affiliations:** ^1^ School of Pharmacy, Jiangxi University of Chinese Medicine, Nanchang, China; ^2^ Affiliated Hospital of Inner Mongolia Minzu University, Inner Mongolia Minzu University, Tongliao, China

**Keywords:** *Citrus* species, chalcone-flavanone isomerase, phylogenetic analysis, gene expression pattern, flavanones

## Abstract

*Citrus* fruit contains rich nutrients which is edible and of officinal value. *Citrus* flavanones are widely used in the treatment of cardiovascular and other diseases, and they are a foundational material of Chinese medicine. The chalcone-flavanone isomerase (CHI) plays a key role in flavanone synthesis. Therefore, we comprehensively analyzed *CHI* genes in *Citrus* species. Here, thirty *CHI* genes were identified for the first time in six *Citrus* species, which were divided into *CHI* and *FAP* groups. Evolutionary analysis showed that *CHI* gene members were highly conserved and were an ancient family. All *CsCHI* genes showed the highest expression level after the second physiological fruit-falling period in *C. sinensis*. *CsCHI1* and *CsCHI3* were highly expressed at 50 days after the flowering (DAF) stage in albedo. The expression of *CsFAP2* and *CsCHI3* genes at the 50 DAF stage was 16.5 and 24.3 times higher than that at the 220 DAF stage, respectively. The expression of *CsCHI1*, *CsCHI3,* and *CsFAP2* genes in the peel was higher than that in the pulp, especially in common sweet orange. The *CsCHI3* gene maintained a high expression level in the epicarp and juice sac at all periods. The members of CHIs interacted with chalcone synthase (CHS), flavonol synthase/flavanone 3-hydroxylase (FLS) and naringenin, and 2-oxoglutarate 3-dioxygenase (F3H) to form heterodimers, which might together play a regulatory role and participate in the flavonoid pathway. This study will provide the basis for the selection of flavonoids in plant tissues and periods and fundamental information for further functional studies.

## Introduction

The *Citrus* fruit belongs to the Rutaceae family and is one of the most widely cultivated fruit crops worldwide (Wu et al., 2018). Many researchers believe that citrus originated in Southeast Asia and began to be cultivated 4,000 years ago (Scora, 1975; Gmitter and Hu, 1990; Xu et al., 2013). Globally, the annual output of citrus fruit is more than 120 million tons (FAO statistics, see URLs), which is not only a nutritional source for human health but also rich in medicinal ingredients (Tocmo et al., 2020; Zhao et al., 2020). Vitamin C, as an important part of human nutrition, mainly comes from *Citrus* fruits (Xu et al., 2013). Moreover, phytochemical studies report that the *Citrus* plant has constituents including flavonoids, limonoids, and carotenoids (Zibaee et al., 2020; Addi et al., 2022). *Citrus* extracts were widely used in the treatment of cardiovascular, gastrointestinal, and other diseases and have anti-oxidant, anti-inflammatory, and nerve-protective effects (Szczepaniak et al., 2020; Zibaee et al., 2020; Rao et al., 2021).

Flavonoids are a kind of important secondary metabolites in plants, mainly in the form of glycosides (Winkel-Shirley, 2001; Kumar and Pandey, 2013). Phe and malonyl-coenzyme A form flavonoids through the fatty acid pathway and flavonoids constitute a diversified aromatic molecular family (Winkel-Shirley, 2001). The characteristic of fruit flavanones is that a disaccharidic moiety is connected to the 7 position of aglycone. Narirutin and naringin in grapefruit, hesperidin and narirutin in orange, and eriocitrin in lemon are the most representative flavanones (Peterson et al., 2006; Chanet et al., 2012; Kumar and Pandey, 2013). *Citrus* flavanones positively influence the cardio-metabolic system and prevent cardiovascular disease (Dauchet et al., 2005; Dauchet et al., 2006; He et al., 2006). For example, the positive effects of *Citrus* flavanones on the cardiovascular system are mainly manifested in the reduction of endothelial dysfunction, improvement of vascular function, and lipid level reduction (He et al., 2006; Testai and Calderone, 2017). The beneficial mechanism of *Citrus* flavanones on the cardiovascular system is mainly manifested in the vasodilator activity, anti-ischemic activity, glucose tolerance, and anti-oxidant and anti-inflammatory actions (Testai and Calderone, 2017). In addition, flavanones also have other pharmacological properties, such as anti-aging and anti-tumor activities, anti-oxidation, and immunity regulation (Yin et al., 2019).

Many studies have shown that naringenin plays an important role in the synthesis of flavanones (Yin et al., 2019). Meanwhile, CHI is a key enzyme in the synthesis of naringenin by the isomerization of 4, 2′, 4′, 6′-tetrahydroxychalcone (Shirley et al., 1992). In 1986, the first *CHI* gene was successfully cloned in *Pisum sativum* L. (Mehdy and Lamb, 1987). So far, more than 3,000 nucleotide sequences have been registered on the National Center for Biotechnology Information (NCBI) GenBank, involving 290 species in 71 families (Yin et al., 2019). Among all these species, medicinal plants account for a large proportion, such as *Glycyrrhiza uralensis*, *Ginkgo biloba* L., and *Mirabilis himalaica* (Zhang et al., 2009; Zhao et al., 2012; Lan et al., 2016). *Arabidopsis* studies have shown the function of CHI as a unique enhancer in the flavone pathway (Jiang et al., 2015). The *CHI* gene promotes fruit yellowing in fresh-cut Chinese water-chestnut (He and Pan, 2017). In addition, the study also found that the expression of the *CHI* gene was positively correlated with flavonoid accumulation in plants (Wang et al., 2010; Guan et al., 2014; Guo et al., 2015).

At present, the regulation of flavonoids in the genus *Citrus* mainly focuses on the identification and function of key transcription factors and some enzymes. However, the systematic analysis of the *CHI* gene family in genus *Citrus* has not been reported. Recently, a type IV *CHI* gene was identified in *Citrus reticulata* cv. *Suavissima*, which enhances the accumulation of flavanones and flavones (Zhao et al., 2021). This study further supports the importance of comprehensively identifying and analyzing the *CHI* gene family in *Citrus*, including gene structure, molecular characterizations, molecular evolution, and genes expression patterns. To conclude, this study improves the understanding of *Citrus CHI* genes and provides a reference for selecting tissue and period for flavonoid extraction. This study will be of great significance for further understanding the mechanism of flavonoid synthesis in *Citrus* species.

## Materials and Methods

### Identification and Characterization of Putative CHI Proteins in *Citrus*

The *Citrus* genome and genome annotation files were obtained from the *Citrus* Pan-genome to Breeding Database (CPBD) (Xu et al., 2013; Wang X. et al., 2017; Wang et al., 2018; Huang et al., 2021), including *Citrus clementina*, *Citrus grandis*, *Citrus reticulate*, *Citrus media*, *Citrus ichangensis,* and *Citrus sinensis*. The *Arabidopsis* and rice genome and genome annotation files were downloaded from The *Arabidopsis* Information Resource (TAIR) and Rice Genome Annotation Project (RGAP), respectively (Kawahara et al., 2013; Berardini et al., 2015). *Selaginella moellendorffii* and *Physcomitrium patens* genome files were obtained from Phytozome (Rensing Stefan et al., 2008; Banks et al., 2011; Goodstein et al., 2012).

The chalcone domain proteins were identified from six *Citrus* species using the HMMER software according to the chalcone domain, with a threshold of e-value < e^−5^ (Johnson et al., 2010). The domain of chalcone-flavanone isomerase (CHI) proteins (PF02431) was downloaded from the Pfam database and confirmed by the Swiss-Prot database (El-Gebali et al., 2019; UniProt, 2019). We validated candidate protein sequences again using the SMART database, and removed protein sequences with obvious errors, of length smaller than 150 aa, and/or ≥95% identity (Sun et al., 2015; Letunic and Bork, 2018). The molecular weight (MW) and isoelectric point (pI) of chalcone domain proteins were calculated by using the ExPASy online tool (Bjellqvist et al., 1993). The subcellular localization of chalcone domain proteins was predicted by using the Protein Subcellular Localization Prediction Tool (PSORT) (Peabody et al., 2020).

### Gene Structure and Motif Analyses

The sequences and annotation information of *CHI* genes were obtained from the genome database. We identified the gene structures, including exon, intron, and 3′ UTR and 5′ UTR regions, according to the genome annotation file using the TBtool software (Chen et al., 2020). We retrieved motifs of CHI protein sequences using the Multiple Em for Motif Elicitation (MEME) tool with the following parameters: the motif width was set to 6–50, the motif number was set to 12, and any number of repetitions (Bailey et al., 2015).

### Phylogenetic and Synteny Analyses

The phylogenetic trees were conducted according to neighbor-joining (NJ) and maximum likelihood (ML) methods, respectively (Saitou and Nei, 1987; Jones et al., 1992). To categorize CHIs, six referential *A. thaliana* CHIs were used: AtCHI1 (AT3G55120), AtCHI2 (AT5G66220), AtCHI3 (AT5G05270), AtFAP1 (AT5G66230), AtFAP2 (AT2G26310), and AtFAP3 (AT1G53520) (Berardini et al., 2015). The phylogenetic trees of the NJ method were conducted using MEGA 7.0 with the following parameters: 1,000 bootstrap resampling, pairwise deletion option, and the Jones–Taylor–Thornton (JTT) model. The phylogenetic trees of the ML method were conducted using MEGA 7.0 with the following parameters: 1,000 bootstrap resampling, complete deletion option, and the Jones–Taylor–Thornton (JTT) model (Kumar et al., 2016).

For the purpose of identifying the synteny of *CHI* genes, the genome sequence of *C. grandis* and *C. sinensis* was downloaded on a local server. First, we merged the genomic data corresponding to the two species. The protein sequences were aligned using the BLAST software with the following parameters: e-value ≤ 1e^−5^ and number threads = 10 (Ye et al., 2006). We analyzed the genome-wide synteny using the MCScanX software with alignment significance (E-value < 1e^−5^) (Wang et al., 2012). The gene pairs of synteny were extracted from the collinearity and tandem files. We visualized synteny gene pairs at the whole chromosome level using the R package circlize.

### Expression Pattern Analysis of *CHI* Genes

RNA-seq data for *C. sinensis* were obtained from the NCBI GEO DataSets under accession numbers PRJNA689213 and PRJNA517400 (Feng et al., 2021; Huang et al., 2021). The transcriptome data of pulp and peel contained two types of sweet oranges (Valencia orange and common sweet orange), involving six varieties. The Valencia orange included “Rohde Red Valencia,” “Delta Valencia,” and “Cutter Valencia” oranges. The common sweet orange included “Xianfeng,” “Jincheng,” and “Taoye” oranges (Huang et al., 2021). Compared with the common sweet orange, Valencia orange belonged to late-ripening sweet oranges which had poor mastication traits (Wu et al., 2020). The transcriptome data of fruit development included four tissues (albedo, epicarp, juice sac, and segment membrane), involving four periods (the second physiological fruit-falling period, the expansion period, the coloring period, and the full-ripening period), which were divided into six time points (Feng et al., 2021). We extracted the expression of CHI genes and analyzed the expression pattern using the R package pheatmap.

To further verify the specific expression of genes, we detected the relative expression of *CsCHI* genes in fruit tissue by real-time quantitative PCR. The isolation of total RNA and the construction of the cDNA library were carried out using the TaKaRa kit (Code No. 9767 and Code No. RR047A). Specific primers of *CsCHI* genes for qRT-PCR were designed using Primer3Plus tools (Supplementary Table S1). We calculated the relative expression of *CsCHI* genes using the delta–delta CT method with the actin gene from sweet orange as the reference gene.

### *CHI* Genes Involved in Flavonoid Metabolism Analysis

Based on the genome sequence and annotation file, the protein sequence and annotation information of CHI proteins were extracted by TBtool software (Chen et al., 2020). We predicted protein interactions in the STRING database by homologous sequence alignment (Szklarczyk et al., 2017). Molecular regulatory pathways were analyzed by the Kyoto Encyclopedia of Genes and Genomes (KEGG) (Kanehisa and Goto, 2000). The chemical molecular structure of matter was visualized using the MolView tool (Smith, 1995).

## Results

### Characterization of Chalcone Domain Proteins in *Citrus* Species

By combining BLAST and HMM searches, a total of 30 chalcone domain proteins were identified across six *Citrus* species (Table 1). Then, each putative protein was assigned to their closest *Arabidopsis* orthologous proteins and named (Supplementary Figure S1; Table 1). In total, this *Citrus* chalcone domain included 13 *CHI* and 17 fatty acid-binding protein (*FAP*) genes (Table 1). We obtained five *CHI* genes in each species, but *C. clementina* contained two *CHI3* (*CcCHI3;1* and *CcCHI3;2*) genes without *FAP1* genes. The sequence lengths varied between 169 and 640 amino acids (aa), the isoelectric point (pI) ranged from 4.81 to 9.23, and the molecular weight (MW) varied from 18.98 to 70.85 kDa (Table 1). Subcellular localization prediction results showed that all CHI subfamily members were predicted to be targeted to the cytoplasm, whereas, CrCHI1, CmCHI1, and CgCHI1 were predicted to be located in the cytoplasm and nucleus, and CcCHI3; 2 was predicted to be located in the cytoplasm and mitochondria (Table 1).

**TABLE 1:** Characteristics of the *CHI* genes identified in *Citrus*.

Name	Gene ID	Locus	Protein length (aa)	MW (kDa)	pI	Localization	Species
*CcCHI1*	Ciclev10032697m	Ciclev10032697m	222	23.98	5.04	Cytoplasm	*Citrus clementina*
*CcCHI3;1*	Ciclev10032749m	Ciclev10032749m	209	23.23	5.01	Cytoplasm	*Citrus clementina*
*CcCHI3;2*	Ciclev10032801m	Ciclev10032801m	197	21.85	5.1	Cytoplasm/mitochondria	*Citrus clementina*
*CcFAP2*	Ciclev10008420m	Ciclev10008420m	419	45.93	8.43	Cytoplasm	*Citrus clementina*
*CcFAP3*	Ciclev10021578m	Ciclev10021578m	279	30.08	9.02	Mitochondria	*Citrus clementina*
*CgCHI1*	Cg7g005600	Cg7g005600	640	70.85	5.68	Cytoplasm/nucleus	*Citrus grandis*
*CgCHI3*	Cg7g003710	Cg7g003710	209	23.32	4.95	Cytoplasm	*Citrus grandis*
*CgFAP1*	Cg5g035430	Cg5g035430	283	31.23	8.95	Nucleus	*Citrus grandis*
*CgFAP2*	Cg4g018640	Cg4g018640	419	45.96	8.43	Cytoplasm	*Citrus grandis*
*CgFAP3*	Cg5g015710	Cg5g015710	188	20.52	6.58	Cytoplasm	*Citrus grandis*
*CiCHI1*	Ci123750	scaffold_98	498	55.13	5.01	Cytoplasm	*Citrus ichangensis*
*CiCHI3*	Ci070860	scaffold_40	209	23.23	5.01	Cytoplasm	*Citrus ichangensis*
*CiFAP1*	Ci208590	scaffold_290	283	31.15	8.96	Nucleus	*Citrus ichangensis*
*CiFAP2*	Ci086900	scaffold_54	419	45.90	8.58	Cytoplasm	*Citrus ichangensis*
*CiFAP3*	Ci157120	scaffold_155	279	30.07	9.14	Mitochondria	*Citrus ichangensis*
*CmCHI1*	Cm154640	scaffold_243	608	67.18	5.3	Cytoplasm/nucleus	*Citrus media*
*CmCHI3*	Cm078090	scaffold_79	209	23.11	5.09	Cytoplasm	*Citrus media*
*CmFAP1*	Cm230370	scaffold_554	282	31.09	8.8	Nucleus	*Citrus media*
*CmFAP2*	Cm086460	scaffold_94	419	45.95	8.43	Cytoplasm	*Citrus media*
*CmFAP3*	Cm084050	scaffold_90	279	30.17	9.14	Mitochondria	*Citrus media*
*CrCHI1*	MSYJ042510	scaffold86082_cov97	608	67.04	5.24	Cytoplasm/nucleus	*Citrus reticulata*
*CrCHI3*	MSYJ145200	scaffold86030_cov92	209	23.23	5.01	Cytoplasm	*Citrus reticulata*
*CrFAP1*	MSYJ218720	scaffold132_cov94	282	31.03	8.96	Nucleus	*Citrus reticulata*
*CrFAP2*	MSYJ122560	scaffold294_cov92	265	29.47	7.57	Mitochondria	*Citrus reticulata*
*CrFAP3*	MSYJ007070	scaffold835_cov91	279	30.14	9.14	Mitochondria	*Citrus reticulata*
*CsCHI1*	Cs7g28130	Cs7g28130	222	23.98	5.04	Cytoplasm	*Citrus sinensis*
*CsCHI3*	Cs7g29780	Cs7g29780	169	18.98	4.81	Cytoplasm	*Citrus sinensis*
*CsFAP1*	Cs5g31220	Cs5g31220	223	24.98	8.9	Mitochondria	*Citrus sinensis*
*CsFAP2*	Cs4g06290	Cs4g06290	419	45.93	8.43	Cytoplasm	*Citrus sinensis*
*CsFAP3*	Cs5g13060	Cs5g13060	279	30.12	9.23	Mitochondria	*Citrus sinensis*

**FIGURE 1:**
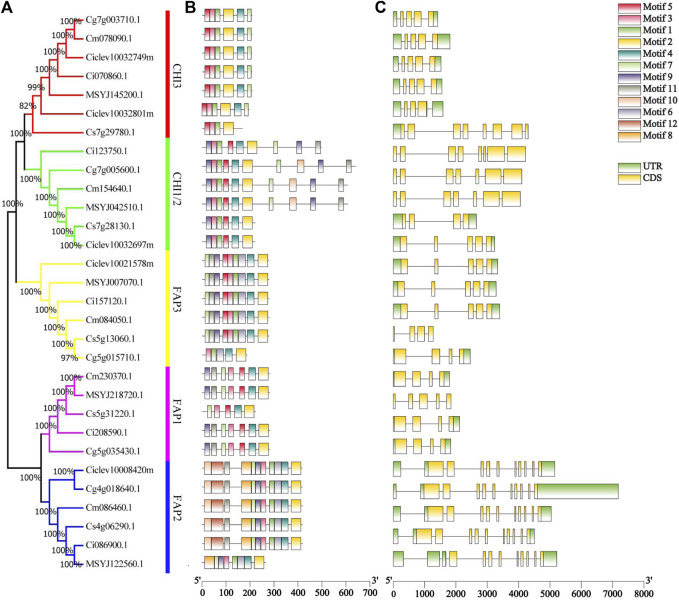
Characterization of 30 *Citrus* chalcone domain proteins. **(A)** Phylogenetic tree of 30 chalcone domain protein sequences. **(B)** Conserved motifs of chalcone domain proteins. Different colored boxes represent different motifs. **(C)** Gene structures of chalcone domain protein genes. Grey lines represent introns, yellow boxes represent exons, and green boxes represent UTR.

We showed the relationships among the 30 CHI genes in the phylogenetic tree (Figure 1A). These proteins were clustered into five groups, which were similar to the groups of *Arabidopsis* CHI (Supplementary Figure S1). A cluster analysis again verified the differences between *C. clementina* CHI members and other species. The motif analysis showed that 12 conserved motifs were identified in 30 CHI/FAP proteins, and the length of the 12 motifs ranged from 21 to 50 aa (Figure 1B). Motifs 1, 3, and 5 together spread over the chalcone domain of CHI proteins (Figure 1B). Even though CHIs and FAPs had the chalcone domain, their amino acid sequences were not completely consistent. Although most of the FAP2 members shared the 11 conserved motifs, CrFAP2 lacked motifs 6, 10, and 12 (Figure 1B). We found that the members of each group had similar motif characteristics. For example, motifs 5, 3, 1, and 2 joined together and appeared in the CHI3 group, whereas motifs 9, 3, 1, 5, 4, and 2 joined together and appeared in the CHI1 group (Figure 1B). *Citrus CHI* genes had a rather loose gene structure, including introns ranging from 3 to 10 (Figure 1C). Except for CsCHI1 carrying three introns, most of the CHI1 genes had six introns in their genomic DNA. In the FAP2, all the genes possessed numerous introns, with 10 or 11 introns (Figure 1C). Combined with the phylogenetic tree, it was found that the *CHI* genes closely related to evolution had similar exon and intron structures in terms of intron number, location, and exon length (Figure 1).

### Phylogenetic Analysis of *CHI* Genes

**FIGURE 2:**
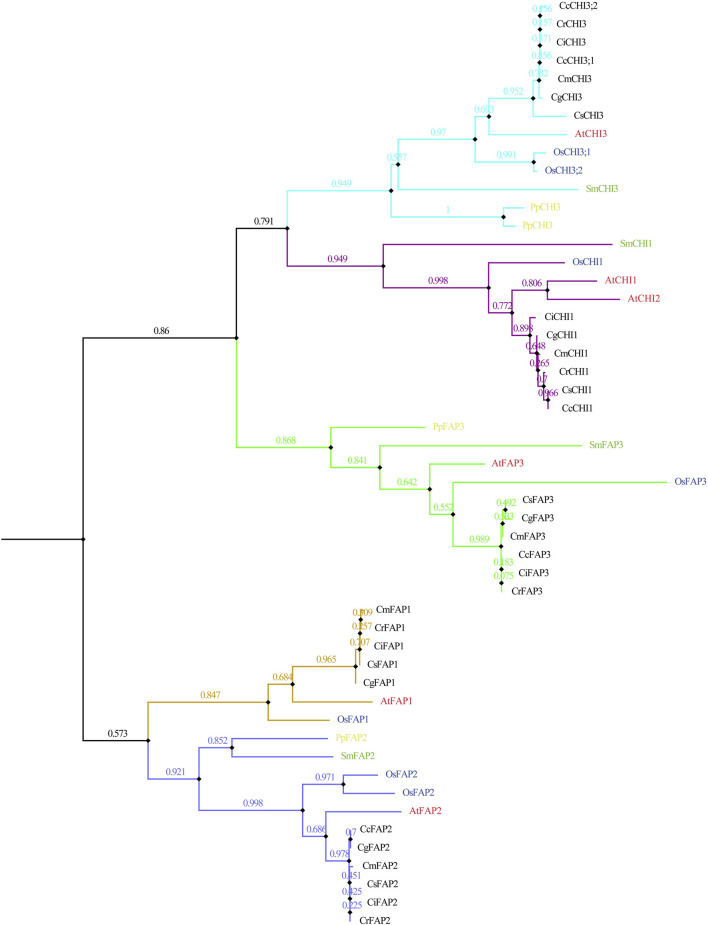
Phylogeny of representative *CHI* genes from the 10 plant species based on the protein sequences. Sm represents *S. moellendorffii,* Pp represents *P. patens*, Os represents *O. sativa*, At represents *A. thaliana*, Cc represents *C. clementina*, Cg represents *C. grandis*, Cr represents *C. reticulata*, Cm represents *C. media*, Cr represents *C. reticulata*, and Cs represents *C. sinensis.*

The evolutionary relationships of *CHI* genes were further explored by phylogenetic and syntenic analyses. Fifty two *CHI* genes were obtained in 10 species, including seven dicotyledons (*C. clementina*, *C. grandis*, *C. reticulata*, *C. media*, *C. reticulata*, *C. sinensis,* and *A. thaliana*), monocotyledons (*O. sativa*), lycophyte (*S. moellendorffii*), and moss (*P. patens*) (Figure 2). Here, we identified four, four, and seven *CHI* genes in *P. patens*, *S. moellendorffii,* and *O. sativa*, respectively. The phylogenetic tree showed five clades, including CHI1/2, CHI3, FAP1, FAP2, and FAP3, whereas, the phylogenetic tree of seven dicotyledons strongly supported these subclades (Supplementary Figure S1). Four *S. moellendorffii CHI* genes were distributed to clades CHI1/2, CHI3, FAP2, and FAP3, respectively (Figure 2). Four *P. patens CHI* genes were distributed to clades CHI3, FAP2, and FAP3, respectively (Figure 2). The *CHI* gene might retain more ancient genetic information in plant evolution. We found that the *CHI* genes of angiosperms had a closer relationship in the subclades. Among them, the *CHI* genes of *Citrus* species were more closely related to *A. thaliana* than *O. sativa*. In general, *CHI* genes were a good gene resource for studying plant evolution. Although each plum plant contains five *CHI* genes, the *CcFAP1* gene was missing in *C. clementina*, but two *CHI3* genes (*CcCHI3;1* and *CcCHI3;2*) were added in the CHI3 clades, which might be related to the expansion/contraction event of the gene family in the process of *C. clementina* evolution.

### Synteny Analysis

**FIGURE 3:**
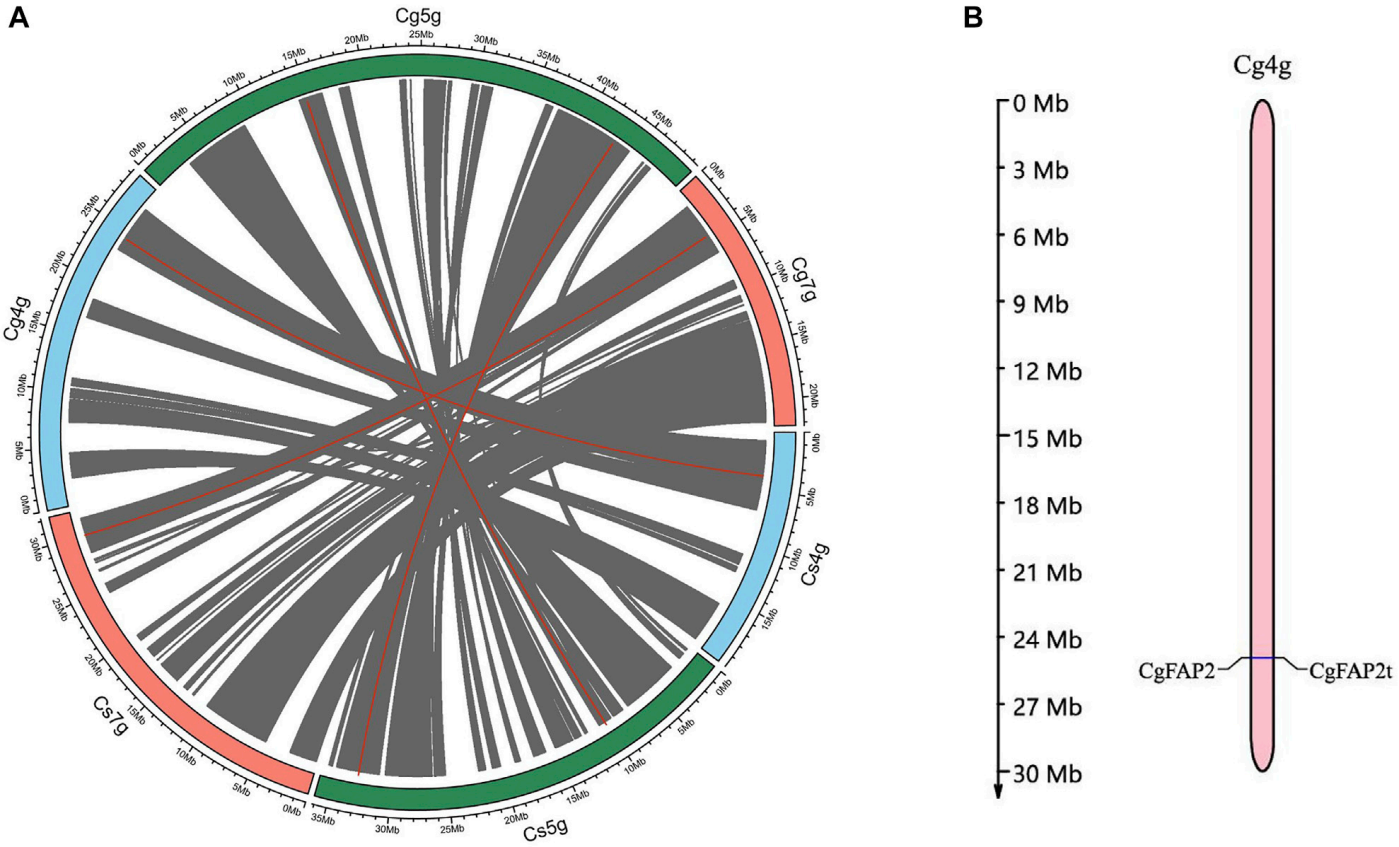
Syntenic analyses of *CHI* genes in *C. grandis* and *C. sinensis*. **(A)** Collinear genes of *C. grandis* and *C. sinensis*. Grey lines represent all collinear genes, red lines represent *CHI* genes between *C. grandis* and *C. sinensis*. **(B)**Tandem genes of *C. grandis CgFAP2* genes. Expression pattern analysis of CHI genes during the fruit development

Based on the genome at the chromosome level of *C. grandis* and *C. sinensis*, we carried out a syntenic analysis of the *CHI* gene. *CgCHI* and *CsCHI* genes were located on chromosomes 4, 5, and 7, respectively. The collinear blocks 11, 22, and 18 containing 4,251 gene pairs were identified on chromosomes 4, 5, and 7, respectively (Figure 3A, Supplementary Table S2). The distribution of the syntenic genes across chromosomes showed that there was an obvious correlation between the chromosomes (Cg4g vs. Cs4g, Cg5g *vs*. Cs5g, and Cg7g *vs*. Cs7g). The checking gene collinearity within a genome showed that 80% of *CHI* genes (*CsCHI3* and *CgCHI3*, *CsFAP3* and *CgFAP3*, *CsFAP2* and *CgFAP2*, and *CsFAP1* and *CgFAP1*) were located in collinear blocks for *C. grandis* and *C. sinensis* (Supplementary Table S3)*.* One tandem duplication was detected for *CHI* genes among *C. grandis* (*CgFAP2* and *CgFAP2t*), which were located on chromosome 4 [(Figure 3B, (Supplementary Table S3)]. These results further proved the close relationship between *C. grandis* and *C. sinensis*.

### Expression Pattern Analysis of CHI Genes During the Fruit Development

**FIGURE 4:**
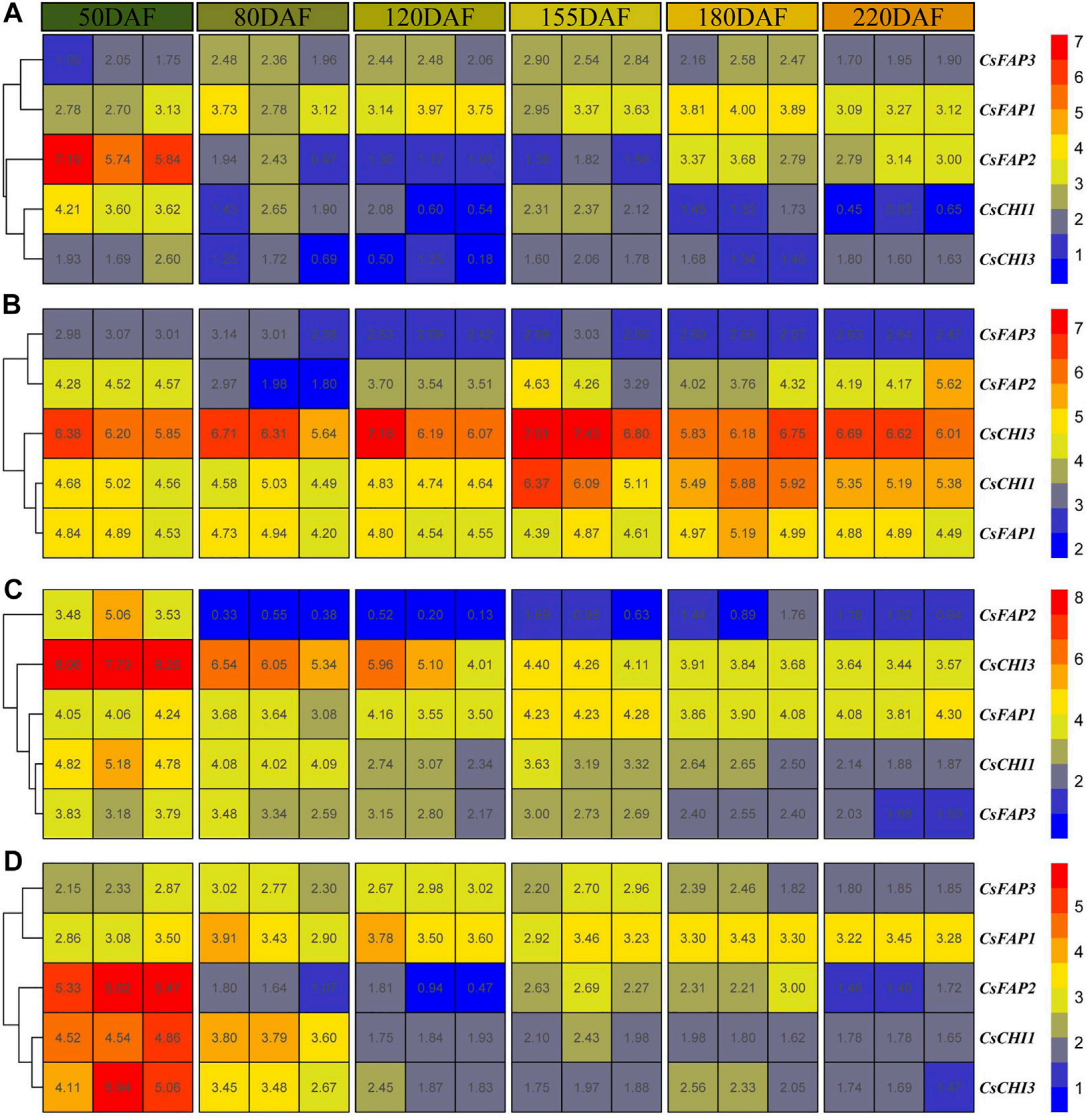
Expression pattern of *CHI* genes during the fruit development. **(A–D)** shows the expression patterns of *CHI* genes in the albedo, epicarp, juice sac, and segment membrane, respectively. 50, 80, 120, 155, 180, and 220 DAF represent 50, 80, 120, 155, 180, and 200 DAF, respectively.

We collected the expression profiles of *C. sinensis* (‘Fengjie 72-1′) albedo, epicarp, juice sac, and segment membrane from six fruit development stages, including 50 days after flowering (the second physiological fruit-falling period); 80, 120, and 155 days after flowering (the expansion period); 180 days after flowering (the coloring period), and 220 days after flowering (the full-ripening period) (Feng et al., 2021). The average expression of all *CsCHI* genes was the highest in the 50 DAF stage, then decreased rapidly, and increased slightly in the 155 DAF stage (Supplementary Table S4). As shown in Figure 4A, *CsCHI1* and *CsCHI3* were highly expressed at the 50 DAF stage in albedo. After that, with the continuous growth and development of fruits, the expression level dropped during the 80 to 220 DAF stage. Surprisingly, the expression level of the *CsFAP2* gene was very high in the 50 DAF stage, which was 12.2 times higher than that in the 220 DAF stage. The *CsFAP3* gene maintained a high expression level in the 80 to 180 DAF stage. As shown in Figure 4B, the expression of CHI genes was higher in the epicarp than in albedo. The expression of *CsCHI1* was upregulated from the 155 DAF stage, and the expression of the *CsFAP2* gene was relatively low at the 80 DAF stage. As shown in Figure 4C, all genes had the highest expression at the 50 DAF stage in the juice sac, and then the expression began to decline, except the *CsFAP1* gene. The expression of the *CsFAP2* gene at the 50 DAF stage was 16.5 times higher than that at the 220 DAF stage. The expressions of *CsFAP2* and *CsCHI3* genes at the 50 DAF stage were 16.5 and 24.3 times higher than that at the 220 DAF stage, respectively. As shown in Figure 4D, five *CsCHI* genes had the highest expression at the 50 DAF stage in the segment membrane, and then the expression began to decline. We found that the expression of the *CsFAP1* gene increased slightly at the 155 and 180 DAF stages. The analysis of gene expression patterns showed that *CsCHI* genes played a major regulatory role at the 50 DAF stage.

### Expression Pattern Analysis of CHI Genes in Different Tissues

**FIGURE 5:**
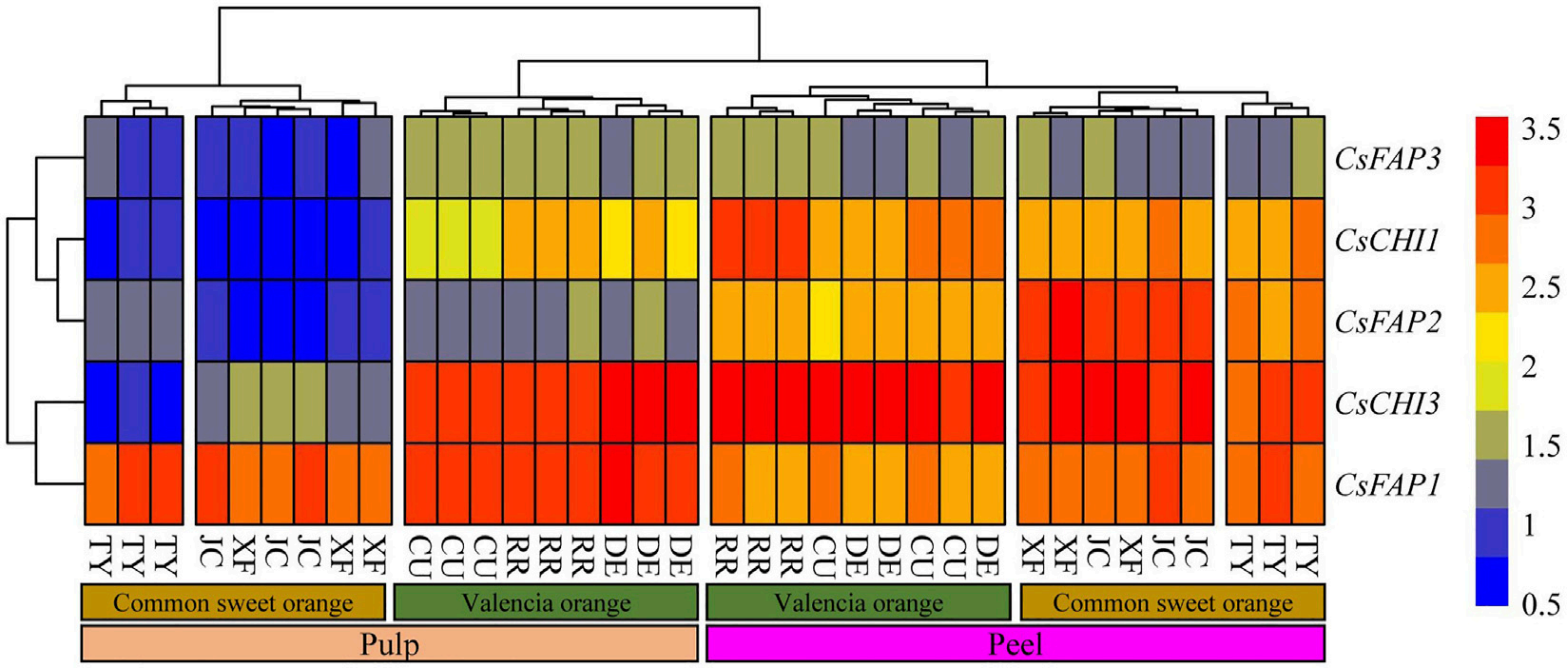
Expression pattern of *CHI* genes in pulp and peel. RR represents “Rohde Red Valencia,” DE represents “Delta Valencia,” CU represents “Cutter Valencia,”, XF represents “Xianfeng,” JC represents “Jincheng,” and TY represents “Taoye” oranges.

To investigate the expression pattern of *CsCHI* genes, 36 samples were used for the expression patterns analysis, including peel and pulp from six varieties based on the available transcriptome data (Huang et al., 2021). The six varieties were selected from two categories: common sweet orange and Valencia orange. The Valencia orange ripened later than the common sweet orange and had a poor mastication trait (Wu et al., 2020). We found that five *CsCHI* genes showed specific expression in the pulp and peel of different varieties (Figure 5, Supplementary Table S5). On the whole, these genes showed higher expression levels in the peel, and the lowest expression levels in the common sweet orange pulp. *CsCHI1* and *CsFAP2* genes with similar expression patterns were clustered into the same subset and specifically expressed in the peel (Figure 5). Compared with pulp, the average expression of *CsCHI1* and *CsFAP2* genes were upregulated by 2.6 and 4.5 times in the peel, respectively. We found that the *CsFAP1* gene was highly expressed in the peel and pulp, and only the *CsFAP1* gene was downregulated in the peel, but the difference was not significant (Figure 5). These results showed that the *CsFAP1* gene had no tissue-specific expression pattern in the peel and pulp. Interestingly, the *CsFAP3* gene was more likely to be upregulated in the three Valencia orange varieties. Meanwhile, the expression of *CsCHI1*, *CsFAP2* and *CsFAP3* genes was hardly detected in the common sweet orange pulp (Figure 5).

**FIGURE 6:**
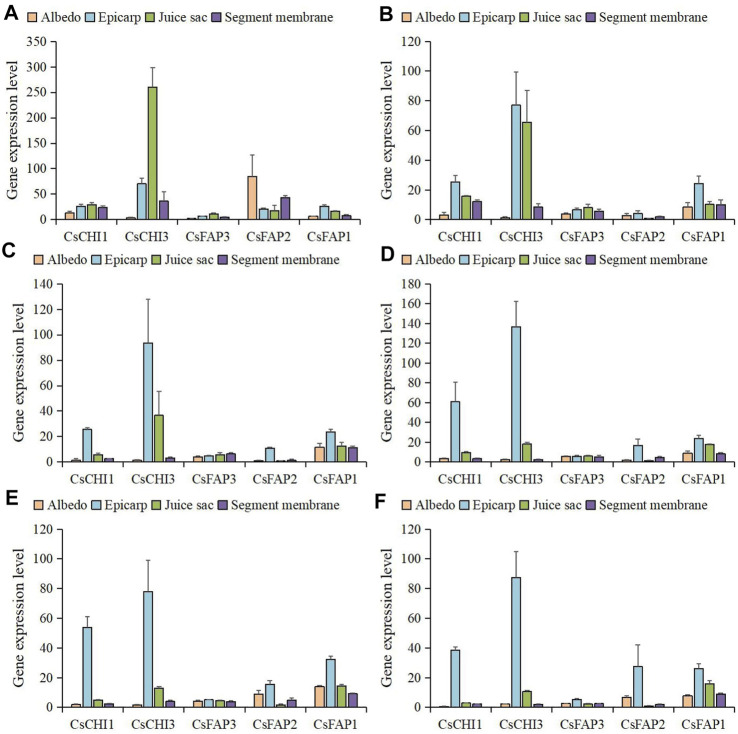
Expression level of *CHI* genes in albedo, epicarp, juice sac, and segment membrane. **(A–F)** shows the expression patterns of *CHI* genes at the 50, 80, 120, 155, 180, and 220 DAF stages.

To further examine the expression level of *CsCHI* genes in different fruit tissues, we compared the expression levels of these genes in the albedo, epicarp, juice sac, and segment membrane (Figure 6; Supplementary Figure S2). We observed that *CsCHI* genes were mainly expressed in the epicarp and juice sac. Interestingly, the expression pattern of the *CsCHI* gene in the epicarp and juice sac showed a negative correlation during fruit development. The *CsCHI3* gene was highly expressed in the epicarp and juice sac, followed by the segment membrane. The expression of the *CsCHI3* gene was hardly detected in the albedo at any developmental stage of the fruit. *CsCHI1* remained highly expressed after the expansion period in the epicarp. Meanwhile, the expression of *CsCHI1* was detected in the albedo at the early stage of fruit development. The expression level of *CsFAP3* was relatively low in four tissues, and there was no obvious tissue-specific expression pattern. *CsFAP1* and *CsFAP2* genes were mainly expressed in the epicarp, especially in the late stage of fruit development.

### Analysis of *CHI* Genes Involved in Flavonoid Metabolism

**FIGURE 7:**
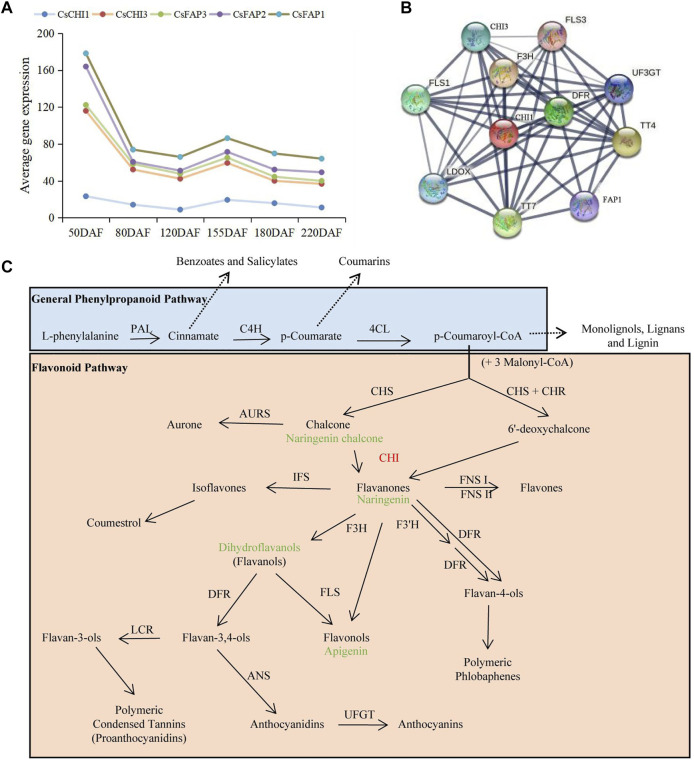
molecular regulation mechanism of *CHI* genes. **(A)** Expression trend of *CHI* genes during the fruit development. **(B)** Interaction networks of the CHI based on their ortholog proteins to *A. thaliana*. **(C)** Flavonoid biosynthetic pathway.

To understand the role of *CHI* genes in the anthocyanin synthesis pathway, we combined the KEGG and STRING databases and previous studies to analyze the molecular regulation mechanism of *CHI* genes. We found that five *CHI* genes had similar gene expression patterns, and there was direct interaction between their expressed proteins (Figure 7A, Supplementary Figure S3; Supplementary Table S6). These results indicated that the part members of *CHI* genes might play a regulatory role together and participated in the regulation of life activities. In addition, the CHIs and TTs, F3H, and FLSs also had strong interactions, and were the key proteins in this interaction network (Figure 7B). TT4, belonging to the chalcone and stilbene synthase family, encoded chalcone synthase (CHS). CHS could catalyze p-coumaroyl-CoA to form naringenin chalcone, which was a key enzyme involved in the biosynthesis of flavonoids. Naringenin chalcone further formed naringenin under the catalysis of CHI. Naringin formed apigenin and dihydrokaempferol under the action of flavonol synthase/flavanone 3-hydroxylase (FLS) and naringenin, 2-oxoglutarate 3-dioxygenase (F3H), respectively (Figure 7C; Supplementary Figure S4). FLS catalyzed the oxidation of both enantiomers of naringenin to give both cis- and trans-dihydrokaempferols. F3H catalyzed the 3-beta-hydroxylation of 2S-flavanones to 2R, 3R-dihydroflavonols which were intermediates in the biosynthesis of flavonols, anthocyanidins, catechins, and proanthocyanidins in plants. The results showed that CHIs played a key role in flavonoid metabolism and were an essential substrate for naringin synthesis.

## Discussion

Flavonoids are composed of many metabolites with different structures, which play a key role in plant growth and development and have important medicinal value (Moore et al., 2014; Testai and Calderone, 2017; Brunetti et al., 2018). Naringenin, an aglycone called naringenin according to its chemical structure, belongs to flavonoids (Szczepaniak et al., 2020). Naringenin produced by CHI is an important precursor of other flavonoids. The *Arabidopsis* genome contains six CHI proteins with the chalcone domain, which are AtCHI1 (AT3G55120), AtCHI2 (AT5G66220), AtCHI3 (AT5G05270), AtFAP1 (AT5G66230), AtFAP2 (AT2G26310), and AtFAP3 (AT1G53520) (UniProt, 2019). In our study, 30 CHI proteins were identified in six *Citrus* species, with five proteins in each species. The CHI2 protein homologous to *Arabidopsis* was missing in all *Citrus* species. AtCHI2 catalyzes the intramolecular cyclization of bicyclic chalcone to tricyclic (s)-flavanone, which has the same function as AtCHI1 (UniProt, 2019). AtCHI1 and AtCHI2 are considered to be two proteins, and the identity of the sequence is only 63.6%. However, the study of chalcone-flavanone isomerase protein genes in *Arabidopsis* shows that *AtCHI2* is a pseudogene (Ngaki et al., 2012). The *CHI* genes of six *Citrus* plants showed that the pseudogene *Citrus CHI2* could not be retained in the process of evolution.

In previous studies, CHI proteins are divided into four types (Type I, II, III, and IV) according to characteristics of the sequence structure (Zhao et al., 2021). Only the CHI proteins of types I and II have enzymatic cyclization activity (Ralston et al., 2005). The CHI of type III (FAP) is the prototype of other CHI-fold proteins, but it has a fatty acid-binding ability (Ngaki et al., 2012). The secondary structure of the CHI of type IV is similar to CHI of type I and II, while the key residues are substituted (Ngaki et al., 2012). The CHI protein of *Arabidopsis* contains three CHI and three FAP proteins (Ngaki et al., 2012). At the same time, the CHI proteins of six *Citrus* species are evenly divided into five subgroups according to the homologues of *Arabidopsis*. Each subgroup had a similar gene structure and conserved motif distribution. These results also further confirmed the functional differences of different types of CHI proteins.

CHI is obviously a lack of related proteins in the primary metabolism of the flavanone pathway (Jez et al., 2000; Jez et al., 2002), which makes the origin of CHI difficult to understand. In 2012, Micheline et al. showed that CHI originated from FAP3 through phylogenetic analysis (Ngaki et al., 2012). Similar results were also confirmed in our CHI phylogenetic tree. The chalcone-binding site of bona fide CHI and key catalytic residues lacked in CHI-like homologues of bacteria and fungi (Gensheimer and Mushegian, 2004). At the same time, some studies showed that *CHI* genes are restricted to vascular plants (Ngaki et al., 2012). The difference in the number of *CHI* genes between non-seed plants and *Citrus* may be the loss or increase of genes caused by species divergence events. The phylogenetic differentiation of CHI is not significant between seed and non-seed plants (*P. patens* and *S. moellendorffii*). The results show that *CHI* genes belong to an ancient family and the *CHI* gene study of soybean has the same conclusion (Ralston et al., 2005).

The level of gene expression directly affects the content of transcripts and the regulation of genes. *GmCHI* genes show root-specific expression in soybean and differential expression by nodulation signals (Ralston et al., 2005). In addition, *LjCHI* genes show differential expression under fungal elicitor treatment in *Lasianthus japonicas* (Shimada et al., 2003). The expression of the *CitCHIL1* gene in flower tissue was higher than that in roots, stems, and leaves in *Citrus reticulata* cv. *Suavissima* (Zhao et al., 2021). This study showed that the expression of *CsCHI1*, *CsCHI3,* and *CsFAP2* genes in peel was higher than that in pulp, especially in the common sweet orange. The *CsCHI3* gene maintained a high expression level in the epicarp and juice sac at all periods. The developing tissue of *Arabidopsis*, including roots, seeds, embryos, cotyledons, tapetum, macrospores, preanthesis, and young seedlings, shows high expression of *FAPs* (Ngaki et al., 2012). The expression of the *AtFAP2* gene can be detected in the whole life cycle. However, *AtFAP1* and *AtFAP3* genes are only expressed in developing and reproductive tissues. These genes have a maximal expression in seeds at 6 DAF (Ngaki et al., 2012). The expression of *CitCHI1*, *CitCHIL1/2,* and *CitFAP1/3* is the highest at 30 DAF, while *CitFAP2* reaches the peak at 120 DAF (Zhao et al., 2021). In this study, all *CHI* genes maintained a high expression level at 50 DAF, and then showed a down-expression trend during fruit development. The expression processing of *CHI* genes in plants is dynamic and characterized by spatio-temporal specificity.

In plants, CHI proteins play essential roles in flavonoid biosynthesis (Mehdy and Lamb, 1987; Winkel-Shirley, 2001; Gensheimer and Mushegian, 2004). The expression pattern of the *CitCHIL1* gene was highly positively correlated with the accumulation of flavonoids, and was highly synchronized with the expression of *CitCHI*, *CitCHS1,* and *CitCHS2* genes (Zhao et al., 2021). The content of flavonoids in the peel is higher than that in pulp (Wang Y. et al., 2017), which is consistent with the expression of *CHI* genes in this study. These results also mean that flavonoid content can be evaluated by detecting the expression of *CHI* and *CHS* genes. The beneficial mechanism of *Citrus* flavanone on the cardiovascular system is mainly manifested in vasodilator activity, anti-ischemic activity, glucose tolerance, and anti-oxidant and anti-inflammatory action (Testai and Calderone, 2017). In addition, flavanones also have other pharmacological properties, such as anti-aging and anti-tumor activities, anti-oxidation, and immunity regulation (Jung et al., 2003; Yamamoto et al., 2008; Testai, 2015; Da Pozzo et al., 2017; Yin et al., 2019). Flavanone compounds are unevenly distributed in fruits, mainly in the albedo and in the membranes separating cloves (Testai and Calderone, 2017). The albedo and membranous parts of *Citrus* fruits are usually discarded in the present processing and eating process. The rational use of Citrus resources can not only produce more valuable products for human beings, but also reduce environmental pollution.

## Conclusion

In conclusion, we comprehensively analyzed the molecular characteristics, gene structure, evolutionary history, expression pattern, and molecular mechanism of *CHI* genes in six *Citrus* species. Thirty *CHI* genes were identified among six *Citrus* species. *Citrus CHI* gene members were highly conserved and are an ancient family. All *CsCHI* genes showed the highest expression level after the second physiological fruit-falling period. *CsCHI1* and *CsCHI3* were highly expressed at the 50 DAF stage in the albedo. The expression of *CsCHI1*, *CsCHI3,* and *CsFAP2* genes in peel was higher than that in the pulp. The *CsCHI3* gene maintained a high expression level in the epicarp and juice sac at all periods. The expression patterns of *CsCHI* genes were analyzed, which provided the basis for the selection of flavonoids in plant tissues and periods. Our study deepens the understanding of the structure and functions of CHIs and extends the knowledge on the transcriptional regulation of flavanones.

## Data Availability

The datasets presented in this study can be found in online repositories. The names of the repository/repositories and accession number(s) can be found at: https://www.ncbi.nlm.nih.gov/, PRJNA689213; https://www.ncbi.nlm.nih.gov/, PRJNA517400.
