# Supplementary material for: Comparative Analysis of the Chalcone-Flavanone Isomerase Genes in Six Citrus Species and Their Expression Analysis in Sweet Orange (Citrus sinensis)
Source: Front Genet. 2022 Apr 12;13:848141. doi: 10.3389/fgene.2022.848141 (PMC9039136; doi:10.3389/fgene.2022.848141)
Supplement: Supplementary file 3 [file Table1.DOCX]

Supplementary Material

# 1 Supplementary Figures and Tables

## 1.1 Supplementary Figures

## **
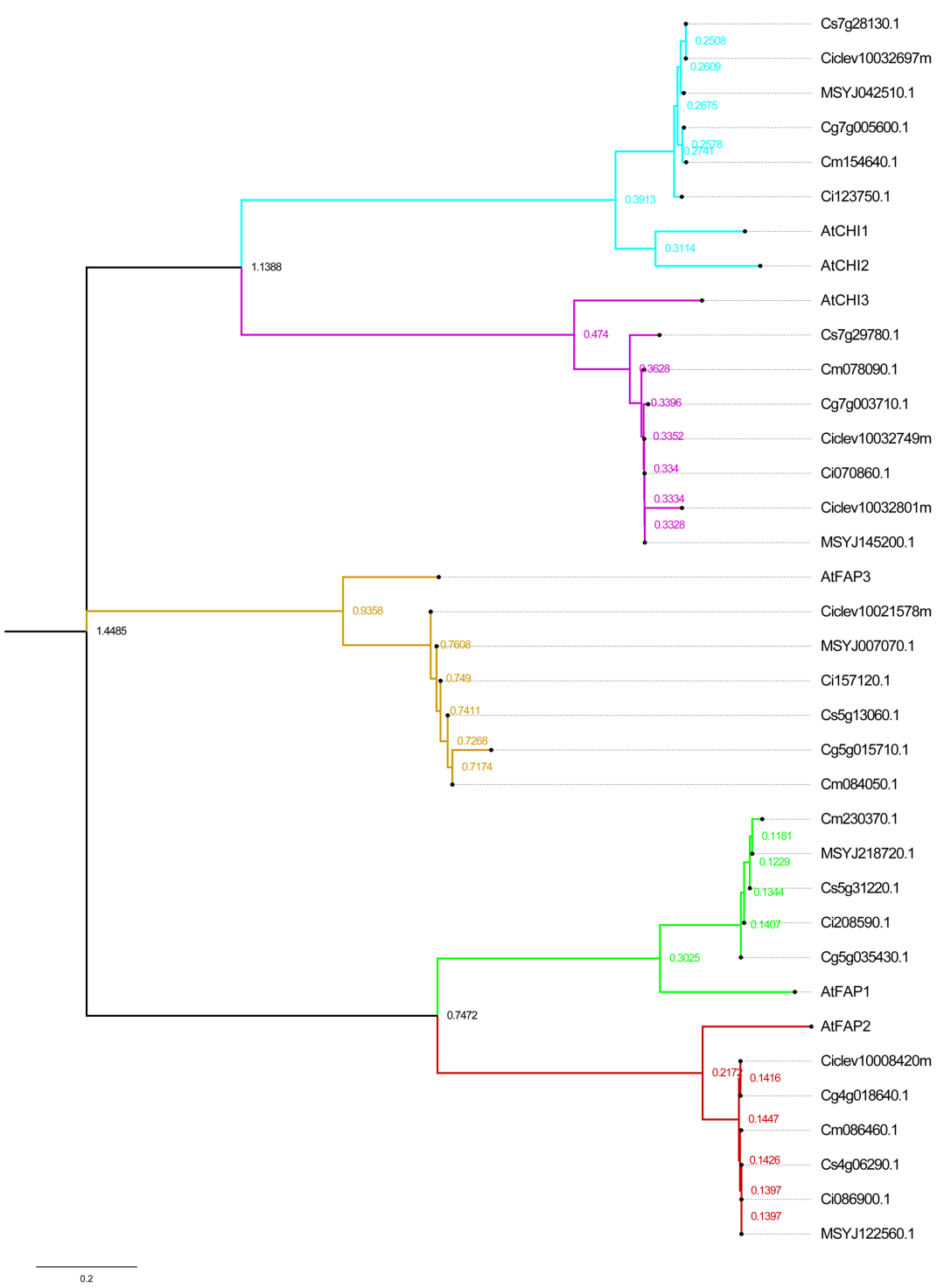
**

## **Supplementary Figure 1.** Phylogeny of representative *CHI* genes from the ten plant species based on the protein sequences.


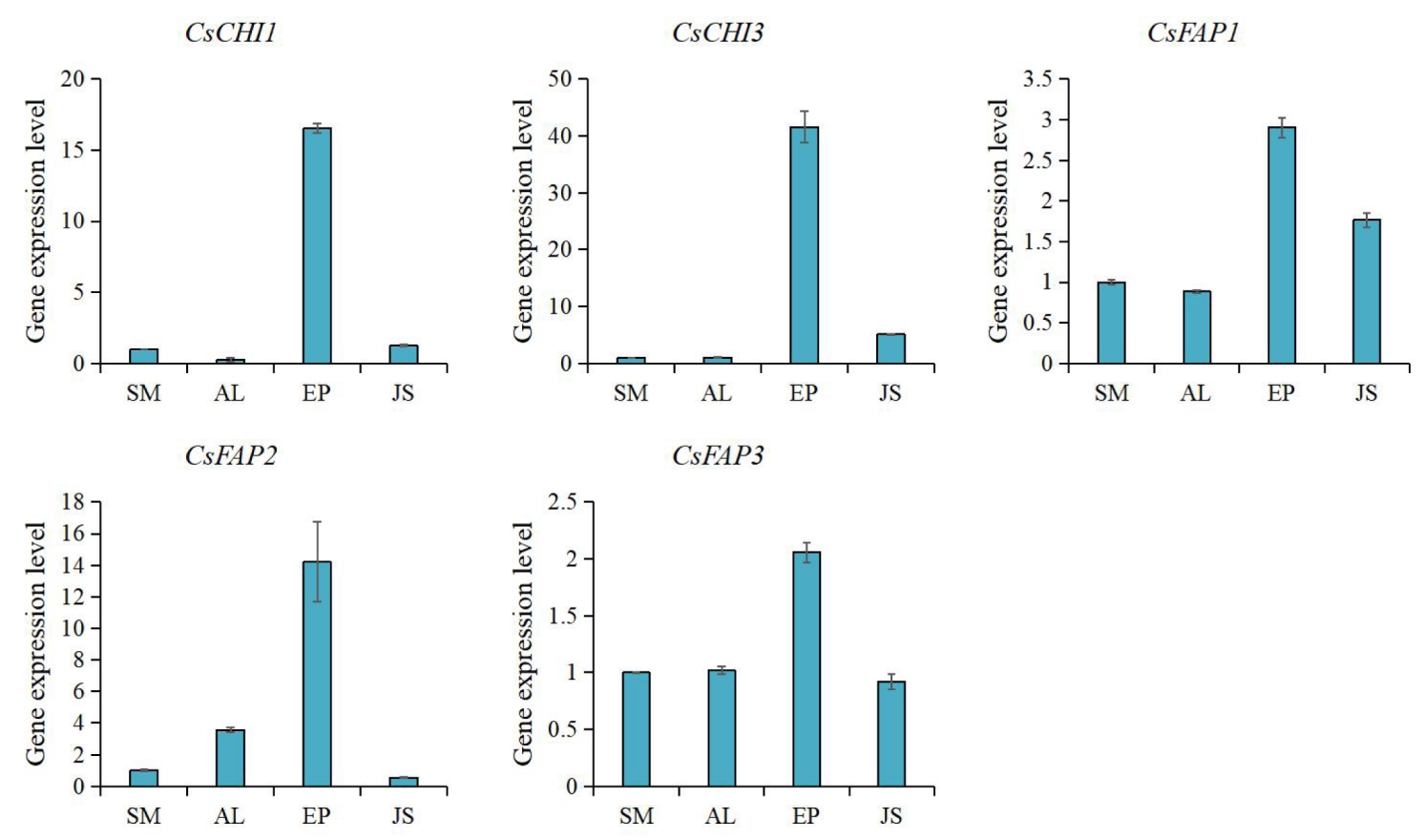


**Supplementary Figure 2.** **Expression level of *CHI* genes in albedo, epicarp, juice sac and segment membrane.**


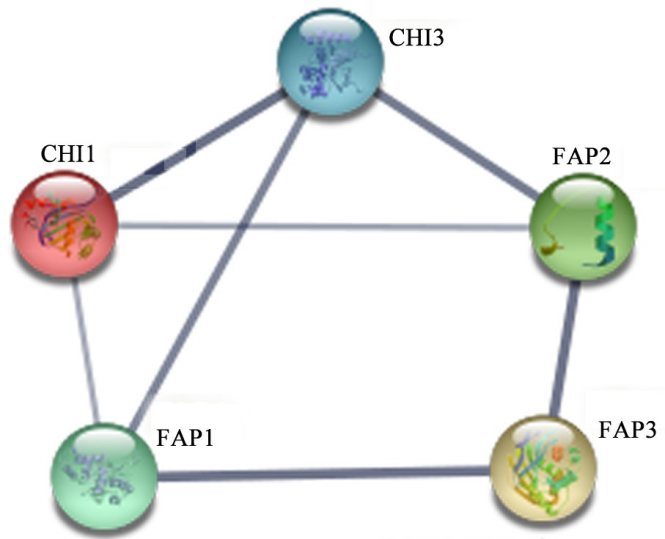


**Supplementary Figure 3.** Interaction networks of the CHI proteins based on their ortholog proteins to *A. thaliana*.


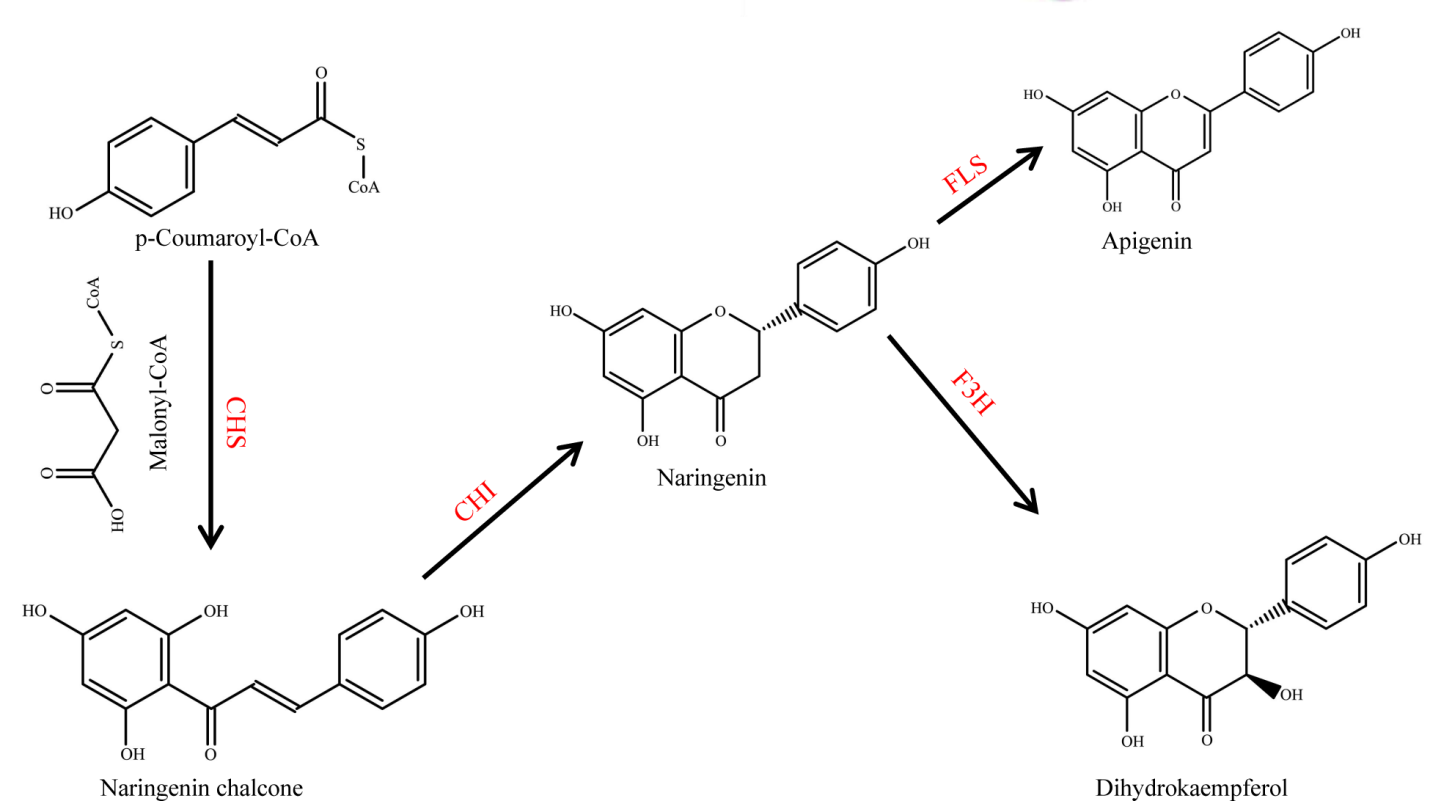


**Supplementary Figure 4. Part of flavonoids biosynthetic pathway.**

## 1.2 Supplementary Tables

**Supplementary Table 1.** Primer sequences used for qRT-PCR

**Supplementary Table 2.** The collinear genes of *C. grandis* and *C. sinensis.*

**Supplementary Table 3.** Syntenic analyses of CHI genes in *C. grandis* and *C. sinensis.*

**Supplementary Table 4.** Expression data of *CHI* genes in pulp and peel.

**Supplementary Table 5.** Expression data of *CHI* genes during the fruit development.

**Supplementary Table 6.** Expression data of *CHS* and *FLS/F3H* genes during the fruit development.
